# Evidence of Experimental Bias in the Life Sciences: Why We Need Blind Data Recording

**DOI:** 10.1371/journal.pbio.1002190

**Published:** 2015-07-08

**Authors:** Luke Holman, Megan L. Head, Robert Lanfear, Michael D. Jennions

**Affiliations:** 1 Division of Evolution, Ecology and Genetics, Research School of Biology, Australian National University, Canberra, Australian Capital Territory, Australia; 2 Department of Biological Sciences, Macquarie University, Sydney, New South Wales, Australia

## Abstract

Observer bias and other “experimenter effects” occur when researchers’ expectations influence study outcome. These biases are strongest when researchers expect a particular result, are measuring subjective variables, and have an incentive to produce data that confirm predictions. To minimize bias, it is good practice to work “blind,” meaning that experimenters are unaware of the identity or treatment group of their subjects while conducting research. Here, using text mining and a literature review, we find evidence that blind protocols are uncommon in the life sciences and that nonblind studies tend to report higher effect sizes and more significant *p*-values. We discuss methods to minimize bias and urge researchers, editors, and peer reviewers to keep blind protocols in mind.

## Abstract 2

Most experiments should ideally be conducted "blind," to avoid introducing bias. A survey of thousands of studies reveals stronger effect sizes and more significant *p*-values in nonblind papers, suggesting that blinding should not be neglected.

## Introduction

Scientific progress rests on reliable data, yet data collection is often subjective. Subjectivity can create biases, many of which derive from cognitive and sensory biases common to us all. For instance, confirmation bias ensures that we preferentially detect, focus on, and recall outcomes that confirm prior beliefs [1]. This tendency causes observer bias during research, whereby the outcome recorded is influenced by the data collector’s beliefs [2–4]. For example, an ornithologist might perceive increased aggression in birds given a testosterone implant, or a psychiatrist could conclude that his or her patient’s mood has improved after prescribing a new antidepressant. Researchers’ expectations can also influence study outcome at other stages of the study besides data collection. For example, researchers may unintentionally treat subjects differently based on their treatment group, such as by providing more food to control group animals or presenting different nonverbal cues to patients in the intervention group of a clinical trial (thus causing placebo/nocebo effects).

Observer bias and other related biases that are collectively known as experimenter effects are greatly minimized if the subjects’ identities are hidden from researchers, and so researchers often employ “blind” protocols when performing experiments and recording data (e.g., [2,5,6]). Working blind means that the subjects’ treatment assignments, and ideally the purpose and expected outcome of the study, are unknown to the experimenter (e.g., he/she is unaware which birds received testosterone or which patients received placebos). Working “double blind” usually refers to concealing treatment assignments from both the subjects and the experimenters, which is often necessary if the subjects are humans (e.g., to standardize placebo effects across treatments). The phrase “single blind” usually means that the subjects, but not the data collectors, were blinded, and thus indicates that the study outcome may have been influenced by experimenter effects such as observer bias [3].

Multiple lines of evidence suggest that observer bias affects data quality. An early experiment showed that students who believed their test rats had been selectively bred for maze-solving ability recorded better maze performance than did students told the rats were bred for poor maze-solving ability, despite both groups possessing randomly assigned, normal rats [7]. Abundant evidence for observer bias has also been obtained from literature surveys. Hróbjartsson et al. [8–10] conducted three meta-analyses of clinical trials in which the same patients had been assessed by both blind and nonblind researchers. Nonblind data collectors reported a substantially more beneficial effect of the clinical intervention than did blind assessors. A number of meta-epidemiological studies comparing clinical or animal model trials that used blind versus nonblind data collection have similarly found evidence that observer bias inflates effect sizes (e.g., [11–15]). There are also counter examples in which blind data collection had no statistically significant effect [16–18].

Despite strong evidence of its importance, blind data recording is often neglected, and its use appears to vary between scientific disciplines. For example, across 960 empirical studies in five animal behavior journals, 6.3% of the sampled studies were conducted blind [19]. Of these, papers published in biology journals or originating from biology/zoology departments were less likely to be blind than those from psychology departments or published in psychology journals, suggesting interdisciplinary differences in training, peer review, or scientific conventions regarding blind data recording. Even in medical research, in which the double-blind randomized controlled trial is widely regarded as the gold standard, blind data recording is not ubiquitous. In a sample of 234 meta-analyses of clinical trials, 33% of the meta-analyses did not contain any double-blind studies [11]. Among 2,220 medical experiments on animals, the data collector was blinded in 24% of studies [20].

The frequency of blind data recording is largely undocumented outside of biomedical and behavioral research, and its importance to data quality has only been demonstrated for clinical and medical research [8–12,17,21] and behavioral science [3,4,22–24]. The fact that blind protocols are not often discussed outside these fields suggests that they might be rare in other areas of the life sciences, though data are lacking. Additionally, it is unclear whether blind protocols are equally essential to data quality in other areas of the life sciences. We thus have two aims: (1) to quantify the frequency of blind protocols in multiple journals and disciplines spanning the life sciences and (2) to assess the impact of blind data recording on research outcomes in multiple disciplines.

We predict that experiments that were not performed blind will tend to report larger effect sizes and stronger rejections of the null hypothesis (i.e., smaller *p*-values, all else equal), because researchers presumably hope to find a statistically significant effect more often than not (cf. [25]). Additionally, we predict that papers that were not conducted blind will contain a higher frequency of significant results (i.e., those with a *p*-value below the standard, arbitrary threshold of *p* = 0.05), for two reasons. Firstly, the aforementioned effect of observer bias on measurements of effect size will increase the proportion of results that reach statistical significance. Secondly, various forms of “*p*-hacking” (reviewed in [26]), in which researchers introduce bias that ensures their results become statistically significant, are much easier if one is not blind. For example, “data peeking,” a common [27] form of malpractice in which one checks the results throughout data collection and then stops collecting data if the results become statistically significant [28], is only possible if one knows the identity of the samples. Working blind also hinders the selective exclusion of outliers, which is another common method of ensuring significant results.

To test these predictions, we used two complementary approaches: a comparison of matched pairs experiments examining similar questions in the field of evolutionary biology and a large-scale text-mining exercise that compared *p*-values retrieved from thousands of life science papers putatively with or without blind protocols. The former approach minimizes the extraneous differences between blind and nonblind studies, giving us a better estimate of the relationship between blindness and effect size, but is too labor intensive to gather data on a large scale. The latter approach provides a less precise measure of the effect of blindness on research results, e.g., because it has minimal ability to correct for confounding differences between blind and nonblind papers, but it allowed us to sample very many papers.

### Comparing the Effect Sizes of Blind and Nonblind Studies

Pilot studies revealed that blind studies were rare in evolutionary biology, precluding use of “meta-meta-analysis” (e.g., [11,12,15]), so we devised a novel approach. We assembled 93 closely matched pairs of publications, each of which contained an otherwise-similar blind and nonblind experiment. After blindly extracting effect sizes from these studies, we tested whether nonblind studies produced larger effect sizes, as predicted if observers’ expectations bias research outcomes.

Of the 93 pairs of studies we identified, ten pairs were discarded because insufficient data were presented to extract an effect size from at least one study in the pair. Within each pair, the effect size Hedges’ *g* of the nonblind study was 0.55 ± 0.25 (mean ± SE) higher than that of the blind study (median difference: 0.38). By convention, effect sizes of *g* > 0.5 are considered large [29], so this difference is striking. Additionally, the nonblind study had the higher effect size in 53/83 pairs, i.e., 63% (95% CIs: 52%–74%) rather than 50% as predicted under the null (binomial test: *p* = 0.015).

**Fig 1:**
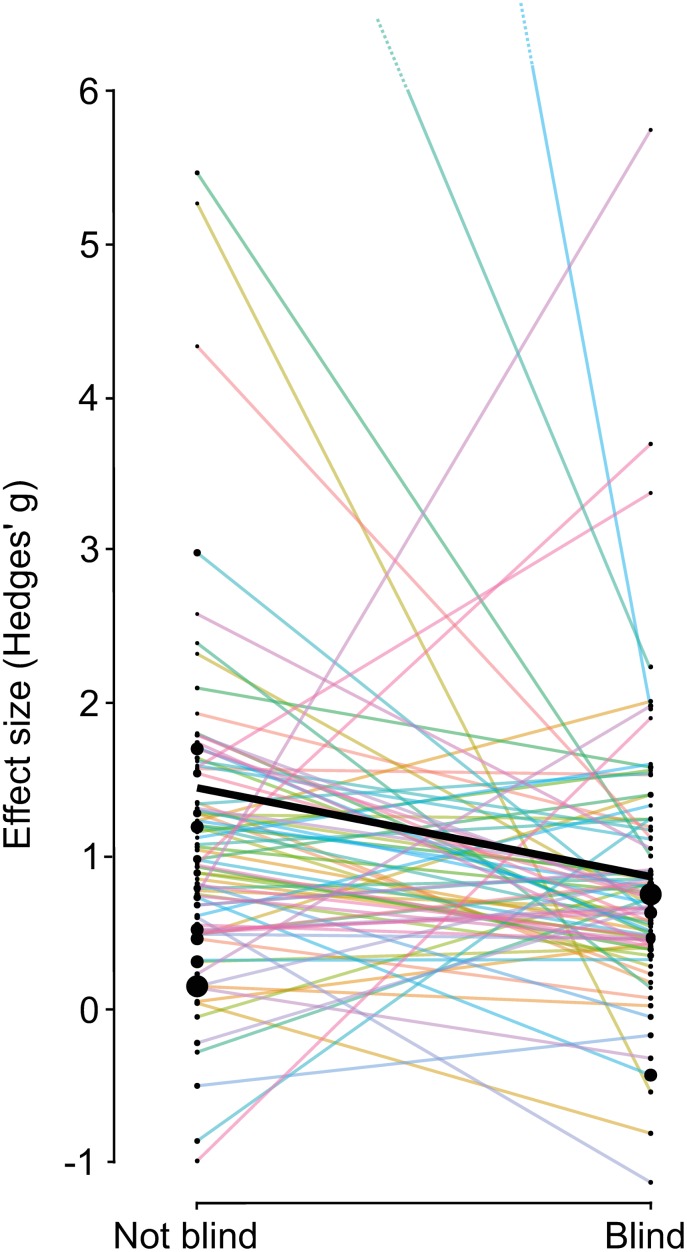
Nonblind studies had higher effect sizes than their paired blind studies, on average (*n* = 83 pairs). The thin lines show individual pairs, while the thick line shows the average effect size for each study type. The size of the dots is inversely proportional to the variance of the effect size, such that larger dots indicate more precise estimates. For clarity, two unusually large effect sizes are off the scale (dotted lines: *g* = 18.0 and 9.1).

We also conducted a formal meta-analysis of these data, which modeled the random variation in effect size between study pairs as well as the random slope of the effect of blindness within study pairs. We included year of publication and the number of authors (log transformed) for each paper as moderator variables, since our text-mining analyses (see below) indicated that these parameters might confound estimation of the effect of blindness. The best model (determined by the corrected Akaike information criterion, AICc) contained the moderators blindness and log author number. The meta-analysis confirmed that blind studies had significantly smaller effect sizes (effect of blindness: *g* = -0.29, z = -2.15, *p* = 0.032) after controlling for the nonsignificant positive correlation between author number and effect size (log author number: *g* = 0.19, z = 1.26, *p* = 0.21). The 95% confidence limits on the effect of blindness were large (-0.025 to -0.55), reflecting the fact that the blind study often, but not always, had the lower effect size within each pair (Fig 1). The grand mean effect size for the papers in our dataset was *g* = 1.17 (95% CIs 0.62 to 1.71, z = 4.2, *p* < 0.0001), meaning that on average, the studies found strong evidence for the effects that had been predicted by their authors (since we defined positive effects as those that went in the predicted direction; see Methods).

Our meta-analysis thus shows that a lack of blindness is associated with an increase in effect size of approximately 27%, i.e., 100 × (−1 + (1.17 + 0.19) / (1.17 + 0.19 − 0.29)). This figure is comparable to estimates from all past meta-analyses on clinical trials of which we are aware. These meta-analyses suggested that a lack of blinding exaggerates the measured benefits of clinical intervention by 22% [11], 25% [12], 27% [10], 36% [8], and even 68% [9].

There was no significant difference in sample size between the blind and nonblind studies, though there was a trend for blind studies to be larger (Mann-Whitney test: W = 599, *p* = 0.054; blind studies: 65.6 ± 14, nonblind studies 38.6 ± 6, *n* = 80 individual papers for which we could identify the sample size).

Additionally, our literature search indicated that blind data recording is rare in evolutionary biology. Our search method involved first identifying a blind study and then searching to find a methodologically similar nonblind study (see Methods). Ideally, we should have struggled to find any nonblind studies, because if the first paper’s authors felt it necessary to work blind, blind data recording should generally have been equally important for researchers doing similar experiments. However, while searching, we found only 0.26 ± 0.05 nonblind papers per blind study. Put another way, the first methodologically comparable paper found for each blind paper was not blind in 73/93 cases.

### Text Mining of Blind and Nonblind *p*-Values

We next used text mining to trawl for experimental studies among an initial list of 870,962 papers from 4,511 journals in the Open Access collection of PubMed, in order to search for signatures of observer bias across the life sciences. After filtering to enrich the list with experimental studies, we used text mining to classify each paper as putatively blind or not blind and extract *p*-values for analysis. After applying our search criteria (S1 Text and S1 Fig), we had 7,644 papers containing at least one *p*-value presented as “*p* =“ (55,688 *p*-values in total) or 12,710 papers for which we could determine the proportion of *p*-values <0.05 (126,060 *p*-values in total). The frequency of papers scored as blind in these datasets was 14.8% and 13.4%, respectively.

**Fig 2:**
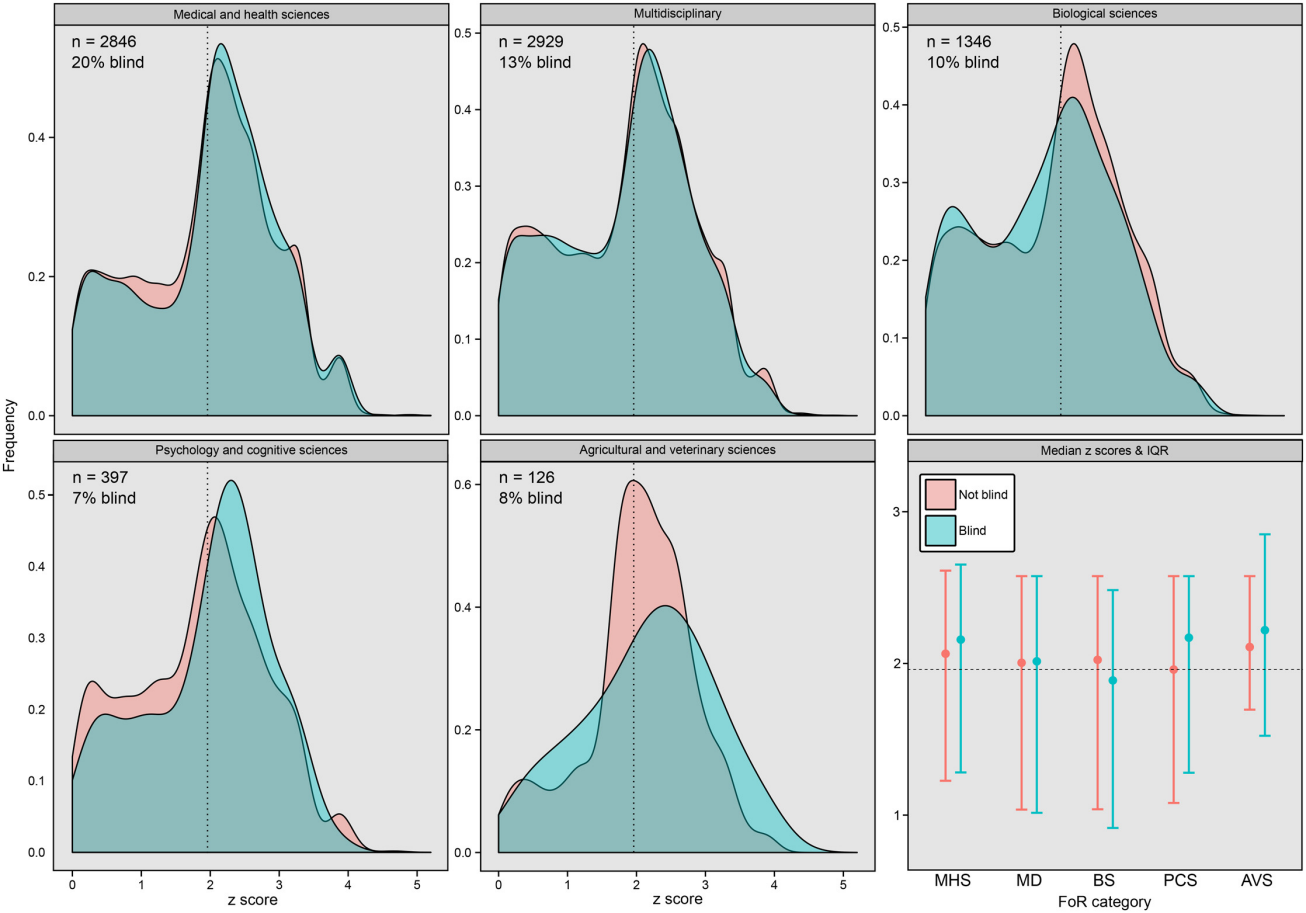
Density plots showing the distribution of z scores taken from putatively experimental blind and nonblind papers. The dotted line shows z = 1.96 (z scores above this line are “significant” at α = 0.05), and the numbers give the sample size (number of papers) and the percentage of papers that were blind for this dataset. The bottom-right figure shows the median z score (and the interquartile range) in each FoR category for blind and nonblind papers.

The analysis of the “*p* =“ dataset is shown in Table 1. The number of authors (both the linear and quadratic terms), year of publication, and scientific discipline (scored objectively using the Field of Research [FoR] journal categorizations of the *Excellence in Research for Australia* initiative) all had a significant effect on z-transformed *p*-values. The estimated effect of blind data recording was small and negative, though its 95% confidence intervals overlapped zero (Table 1; Fig 2). The analysis of the “*p* =“ dataset therefore failed to reject the null hypothesis that the mean *p*-value is the same in our blind and nonblind paper categories. This analysis also showed that z scores increase (i.e., *p*-values decrease) with author number, but the rate of increase slows down as author number grows (S2 Fig). Additionally, z scores have declined (i.e., *p*-values have increased) with time (S3 Fig).

**Table 1:** Effect of each parameter on z scores, as estimated by model averaging of the top four models in the set (those with an Akaike weight > 0.05). Note that the predictor and response variables were rescaled to have a mean of 0 and standard deviation of 0.5 prior to running the models [37]. The FoR category × Blindness interaction was not among the top models, indicating it had no detectable effect on z score. The effects of each FoR category are shown relative to the reference level “Agricultural and veterinary sciences.” The Importance column gives the summed Akaike weights of all the models containing the focal predictor; high importance shows that a parameter was present in many of the best models, and was thus a good predictor of z scores relative to the other measured variables.

Parameter	Estimate	SE	Estimate 95% CIs	Importance
Intercept	2.03	0.061	1.91 to 2.15	
Blindness	-0.025	0.021	-0.059 to 0.024	0.42
Number of authors				
*Linear effect*	0.18	0.018	0.147 to 0.218	>0.99
*Quadratic effect*	-0.032	0.006	-0.044 to -0.019	>0.99
Year of publication	-0.034	0.015	-0.063 to -0.005	0.83
FoR category				
*Biological sciences*	-0.12	0.064	-0.25 to 0.003	>0.99
*Medical and health sciences*	-0.070	0.062	-0.19 to 0.005	
*Multidisciplinary*	-0.15	0.062	-0.27 to -0.025	
*Psychology and cognitive sciences*	-0.15	0.069	-0.28 to -0.011	

**Fig 3:**
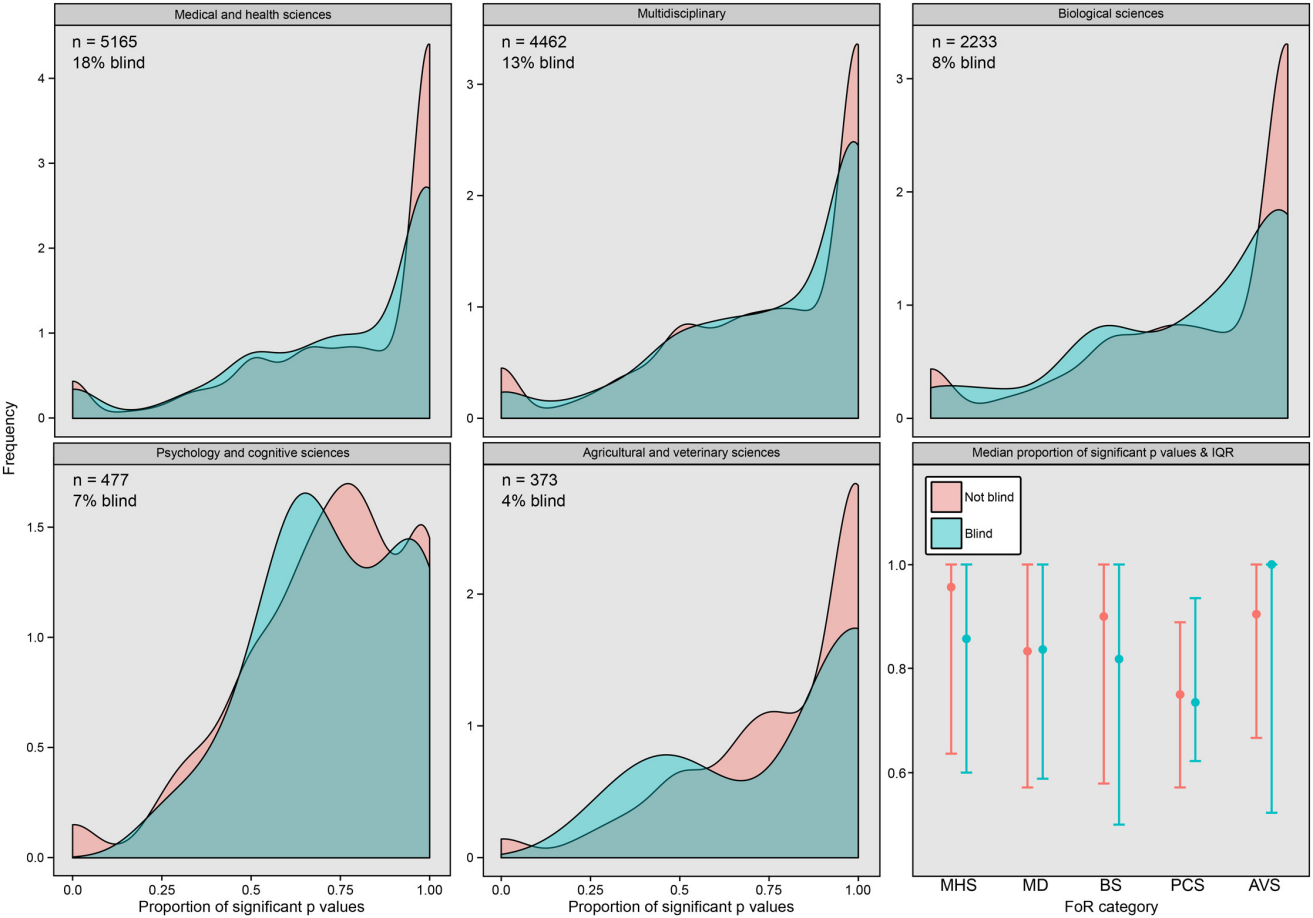
Density plots showing the distribution of the proportion of significant *p*-values per paper (i.e., the number of *p*-values <0.05, divided by the total number of *p*-values) in putatively experimental blind and nonblind papers. The numbers give the sample size (number of papers) and the percentage of papers that were blind for this dataset (note the higher sample size relative to Fig 2). The bottom-right figure shows the median proportion of significant *p*-value papers (and the interquartile range) in each FoR category for blind and nonblind papers.

By contrast, the analysis of the proportion of significant *p*-values in a paper suggested that blindness, and all other parameters except the blindness × FoR category interaction, had a significant effect on *p*-values (Table 2; Fig 3). The top model (shown in Table 2) contained blindness, the linear and quadratic effects of author number, year published, and FoR category, and it was better than the second-best model (which contained Table 2‘s parameters plus the Blindness × FoR category interaction), with a ΔQAIC score of 4.58 (Akaike weight = 0.91). Blind papers had a significantly lower proportion of significant results (Table 2). The effects of year and number of authors were in the same direction as in the previous analysis, and there were differences in the proportion of significant results among FoR categories (Table 2). The meager gain in information provided by fitting the Blindness × FoR category interaction suggests that blindness had a similarly negative effect on the proportion of significant results across all scientific disciplines (see Fig 3). We also note that the majority of papers presented mostly significant *p*-values across all FoR categories (Fig 3).

**Table 2:** Results of a generalized linear model with the proportion of significant *p*-values in each paper as the response variable and binomial errors. Note that the predictor and response variables were rescaled to have a mean of 0 and standard deviation of 0.5 prior to running the model [37]. The effects of each FoR category are shown relative to the reference level “Agricultural and Veterinary Sciences.” The 95% CIs for each parameter were estimated as ±1.96 * SE.

Parameter	Estimate	SE	Estimate 95% CIs	*t*	*p*
Intercept	1.29	0.07	1.16 to 1.43	18.5	<0.0001
Blindness	-0.12	0.03	-0.18 to -0.061	-3.95	<0.0001
Number of authors					
*Linear effect*	0.42	0.03	0.36 to 0.48	14.3	<0.0001
*Quadratic effect*	-0.085	0.01	-0.11 to -0.058	-6.30	<0.0001
Year of publication	-0.10	0.02	-0.15 to -0.052	-4.09	<0.0001
FoR category					
*Biological sciences*	-0.30	0.08	-0.45 to -0.15	-3.93	<0.0001
*Medical and health sciences*	-0.062	0.07	-0.20 to 0.080	-0.86	0.39
*Multidisciplinary*	-0.28	0.07	-0.42 to -0.14	-3.90	0.0001
*Psychology and cognitive sciences*	-0.28	0.08	-0.44 to -0.12	-3.45	0.0002

Lastly, we used the text-mined data to identify predictors of blindness. We ran a binomial generalized linear model on the dataset containing 12,710 papers, with the blindness of each paper as a binary response variable and author number (both linear and quadratic effects), publication year, and FoR category as predictors. The frequency of blindness varied substantially between FoR categories (Fig 2 and Fig 3; likelihood ratio test *p* < 10^−15^). Papers with more authors were more likely to be blind, though this relationship leveled off for high author numbers (S4 Fig; linear effect: z_12702_ = 9.9, *p* < 0.0001; quadratic effect: z_12702_ = -4.8, *p* < 0.0001). Finally, there was no effect of publication year, suggesting the frequency of blind protocols has not changed since the year 2000 (z_12702_ = 0.58, *p* = 0.57).

In summary, our text mining found some evidence that blind and nonblind papers contain different distributions of *p*-values. Although *p*-values presented as exact numbers (“*p* =“) appeared to be uncorrelated with blindness, the proportion of *p*-values per paper falling below the widely used threshold of *p* = 0.05 was higher by 0.12 in nonblind papers, after controlling for the other predictors. Given that the mean number of *p*-values per paper in this dataset was 9.9 ± 0.10, this result suggests that the nonblind papers contained around 1.2 extra statistical results with *p* < 0.05, on average.

## Discussion

### Findings and Limitations of Our Study

Both the paired studies and the text-mining data suggest that blind data recording is less common than one would hope. It was challenging to find blind evolutionary biology studies but easy to find nonblind studies to pair with them. This is concerning because essentially all of these experiments should have been performed blind. Likewise, our text-mined data provide evidence that blind data recording is often neglected (see percentages in Figs 2 and 3).

More worryingly, some of our analyses found correlations between blindness and research outcomes. The most direct evidence comes from the comparison of paired evolutionary biology studies: meta-analysis indicated that a lack of blind data recording inflates the mean reported effect size by 27% on average. We defined positive effect sizes as those that went in the direction predicted by the authors, suggesting this effect of blindness resulted from observer bias. We note that the effect size increase we estimate for a lack of blinding (*g* ≈ 0.29) is comparable to, or larger than, the effect sizes typically studied by evolutionary biologists, according to recent meta-analyses (e.g., [30–32]).

The text-mining data are in partial agreement with this result. Nonblind papers had a higher frequency of significant *p*-values than blind papers, which provides correlational evidence that the resulting bias artificially increases the probability the null hypothesis will be rejected. Intriguingly, however, an effect of blindness was not detected when looking at only exact *p*-values (i.e., those presented as “*p* =“). There are multiple, nonexclusive reasons why this discrepancy might have arisen, besides the lower sample size and hence statistical power of the “*p* =“ analysis. For example, it is possible that the frequency or effectiveness of *p*-hacking [26] is greater in nonblind studies or that studies containing many results that are near to *p* = 0.05 tend to differ systematically from those containing mostly very small or very large *p*-values, in a manner that affects the importance or frequency of blind protocols. Overall, the text-mining data provide some evidence for an effect of blind protocols on research outcomes.

We reiterate that both our analyses are based on nonexperimental data, so we cannot ascertain whether biases deriving from nonblind protocols have a causal effect. Blind and nonblind studies might differ consistently in some other respect besides the opportunity for bias, such as experimental design, sample size, or scientific discipline, leading to a spurious association between blindness and study outcome. The problem of assigning causality is partially alleviated in our paired comparison, since studies were matched closely, and we compared effect sizes, not *p*-values (mitigating the confounding effect of sample size). We nevertheless suggest that observer bias is probably an important causal effect in both datasets, given abundant experimental (e.g. [3,4,8]) and correlational [8–12] evidence that observer bias is common and strong.

Incidentally, our text-mining data revealed a positive relationship between the number of authors and the significance level of results reported in the paper. Multiauthor papers contained smaller *p*-values and a higher proportion of significant *p*-values. As before, this result has at least three possible, nonexclusive explanations: the true mean effect size in multiauthor papers is higher, multiauthor papers have larger sample sizes, and multiauthor papers are more likely to report significant *p*-values but leave nonsignificant ones unspecified. We also found that more recent studies have larger *p*-values (cf. [33,34]), which could similarly be explained by a decline in true effect sizes being studied over time [35,36], a decrease in average sample size, or changes in how statistics are presented (e.g., increasing insistence by journals that all statistical results, including nonsignificant ones, are reported).

Our text-mining approach facilitates the study of large numbers of papers in a broad range of fields, but it is important to acknowledge its limitations. For example, classifying research as nonblind is difficult to do perfectly using text mining, because there are at least three reasons a paper might lack our search terms (“blind/blinded/blindly”): (1) it contained research that should have been blind, but it was not (these are the papers we want to find); (2) the authors used alternative language to declare they had worked blind (or worked blind without declaring it); or (3) it described research that did not need to be blind, such as a mathematical model or an experiment with an objective response variable. By reading 100 of our putatively nonblind studies (S1 Text), we confirmed that the error in point 2 was rare. We could not reliably assess the frequency of the error from point 3, since many of the papers’ methods were inscrutable. Although we took multiple steps to exclude papers that did not require blind protocols (e.g., by only including papers whose abstracts contained the word “experiment” or related words), it is likely that many such papers remain in the dataset. Crucially, however, these papers decrease our chances of detecting an effect of blind data recording by adding uninformative noise to our model estimates. As a consequence, our text-mining results are probably conservative and so might underestimate the impact of a failure to work blind on research results.

### Recommendations for the Future

Our data, and substantial previous work (e.g. [3,8,11–13,15,21,22,24]), suggest that the scarcity of blind protocols has adversely affected published research. We hope that readers will reflect on how they can reduce experimenter effects in their research and be mindful of this when reviewing and editing manuscripts. While not all data need to be collected blind, we suggest it is usually worth erring on the side of caution and working blind when running experiments, collecting data, and applying statistical tests. In some cases, it is impossible or prohibitively demanding to work blind. Here, authors should declare and discuss the potential for observer bias; it might also be worthwhile to use multiple observers and trust in the “wisdom of crowds” to reduce bias [19].

Meta-analysis can be used to catalog the potential for observer bias in published experiments and assess its putative effects. When collecting effect sizes on a focal research question, one can record whether the primary studies were blind. It is common to discard all nonblind datasets from a meta-analysis or to do the analysis both with and without the nonblind experiments (see Cochrane guidelines, http://handbook.cochrane.org/). One could also keep all studies and include blindness as a moderator variable in the meta-analysis.

In closing, it is time for reviewers, editors, and other assessors to insist on blind methods across the life sciences. We perceive a tendency to regard working blind as an unnecessary nuisance, but the evidence suggests that blind protocols are vital to good research practice.

## Supporting Information

S1 Fig PRISMA flowchart. The flowchart shows how articles were culled from the initial list of 739,451 articles downloaded from PubMed to produce our two datasets.
(TIF)

S2 Fig Relationship between author number and text-mined *p*-values. z scores and the proportion of significant *p*-values per paper increase with the number of authors on a paper, and this relationship levels off for higher author numbers. Both plots show means ± SE (points with no error bars have no replication); for the left panel, we first averaged all the z scores from each paper and then took the average of these for each year. The dashed line shows z = 1.96, which is equivalent to *p* = 0.05. Note that higher z scores denote lower *p*-values.
(TIF)

S3 Fig Relationship between publication year and text-mined *p*-values. z scores and the proportion of significant *p*-values per paper have declined in recent years. Both plots show means ± SE (points with no error bars have no replication); for the left panel, we first averaged all the z scores from each paper and then took the average of these for each year. The dashed line shows z = 1.96. Note that higher z scores denote lower *p*-values.
(TIF)

S4 Fig Relationship between author number and probability a paper was blind. Effect of author number on the number of authors on a paper was positively correlated with the probability that a paper was blind, and this relationship began to level off for higher author numbers. Each point shows the mean ± SE for each number of authors (points with no error bars have no replication), and the line and its 95% CIs are from a quadratic regression on all the data. Note that quadratic regressions must invert at some point, but the paucity of data above c. 20 authors suggests that we have weak evidence that the relationship really does decline (not, for example, plateau) for high author numbers as suggested by the regression fit.
(TIF)

S5 Fig Results of a survey showing that abstracts do not contain much information on effect size. The *x*-axis shows which paper in the study pair had the larger absolute effect size (A and B are random names for the papers). The *y*-axis shows the tendency to think paper A had the larger effect, which was calculated as ((Number of participants thinking A was larger)–(Number thinking B was larger)) / 9. Therefore, a score of 1 means that 100% of participants thought A was larger, a score of -1 means that 100% thought B was larger, and zero means that the answers were evenly split. The distribution of answers was not affected by the true effect size difference of the papers (Mann-Whitney test: W = 1011, *p* = 0.17), and there are many study pairs in which the participants either disagreed about the answer or guessed incorrectly en masse.
(TIF)

S1 Text Supplementary methods. (DOCX)

## References

[1] Nickerson RS (1998) Confirmation bias: A ubiquitous phenomenon in many guises. Review of General Psychology 2: 175–220.

[2] Rosenthal R (1966) Experimenter Effects in Behavioral Research. East Norwalk, CT: Appleton-Century-Crofts.

[3] Rosenthal R (2009) Artifacts in Behavioral Research. Oxford: Oxford University Press.

[4] Rosenthal R (1994) Interpersonal expectancy effects: A 30-year perspective. Current Directions in Psychological Science 3: 176–179.

[5] Schulz KF, Grimes DA (2002) Blinding in randomised trials: hiding who got what. Lancet 359: 696–700. 11879884 10.1016/S0140-6736(02)07816-9

[6] Kilkenny C, Parsons N, Kadyszewski E, Festing MFW, Cuthill IC, et al (2009) Survey of the quality of experimental design, statistical analysis and reporting of research using animals. PLoS ONE 4: e7824 10.1371/journal.pone.0007824 19956596 PMC2779358

[7] Rosenthal R, Fode KL (1963) The effect of experimenter bias on the performance of the albino-rat. Behavioral Science 8: 183–189.

[8] Hróbjartsson A, Skou Thomsen AS, Emanuelsson F, Tendal B, Hilden J, et al (2012) Observer bias in randomised clinical trials with binary outcomes: systematic review of trials with both blinded and non-blinded outcome assessors. BMJ 344: e1119 10.1136/bmj.e1119 22371859

[9] Hróbjartsson A, Thomsen ASS, Emanuelsson F, Tendal B, Hilden J, et al (2013) Observer bias in randomized clinical trials with measurement scale outcomes: a systematic review of trials with both blinded and nonblinded assessors. Canadian Medical Association Journal 185: E201–E211. 10.1503/cmaj.120744 23359047 PMC3589328

[10] Hróbjartsson A, Thomsen ASS, Emanuelsson F, Tendal B, Rasmussen JV, et al (2014) Observer bias in randomized clinical trials with time-to-event outcomes: systematic review of trials with both blinded and non-blinded outcome assessors. Int J Epidemiol 43: 937–948. 10.1093/ije/dyt270 24448109

[11] Savović J, Jones HE, Altman DG, Harris RJ, Jüni P, et al (2012) Influence of reported study design characteristics on intervention effect estimates from randomized, controlled trials. Ann Intern Med 157: 429–438. 22945832 10.7326/0003-4819-157-6-201209180-00537

[12] Wood L, Egger M, Gluud LL, Schulz KF, Juni P, et al (2008) Empirical evidence of bias in treatment effect estimates in controlled trials with different interventions and outcomes: meta-epidemiological study. BMJ 336: 601–605. 10.1136/bmj.39465.451748.AD 18316340 PMC2267990

[13] Schulz KF, Chalmers I, Hayes RJ, Altman DG (1995) Empirical evidence of bias. Dimensions of methodological quality associated with estimates of treatment effects in controlled trials. JAMA 273: 408–412. 7823387 10.1001/jama.273.5.408

[14] Colditz GA, Miller JN, Mosteller F (1989) How study design affects outcomes in comparisons of therapy. I: Medical. Stat Med 8: 441–454. 2727468 10.1002/sim.4780080408

[15] Bello S, Krogsbøll LT, Gruber J, Zhao ZJ, Fischer D, et al (2014) Lack of blinding of outcome assessors in animal model experiments implies risk of observer bias. J Clin Epidemiol 67: 973–983. 10.1016/j.jclinepi.2014.04.008 24972762

[16] Hirst JA, Howick J, Aronson JK, Roberts N, Perera R, et al (2014) The need for randomization in animal trials: an overview of systematic reviews. PLoS ONE 9: e98856 10.1371/journal.pone.0098856 24906117 PMC4048216

[17] Crossley NA, Sena E, Goehler J, Horn J, van der Worp B, et al (2008) Empirical evidence of bias in the design of experimental stroke studies: a metaepidemiologic approach. Stroke 39: 929–934. 10.1161/STROKEAHA.107.498725 18239164

[18] Macleod MR, van der Worp HB, Sena ES, Howells DW, Dirnagl U, et al (2008) Evidence for the efficacy of NXY-059 in experimental focal cerebral ischaemia is confounded by study quality. Stroke 39: 2824–2829. 10.1161/STROKEAHA.108.515957 18635842

[19] Burghardt GM, Bartmess-LeVasseur JN, Browning SA, Morrison KE, Stec CL, et al (2012) Minimizing observer bias in behavioral studies: a review and recommendations. Ethology 118: 511–517.

[20] van Luijk J, Bakker B, Rovers MM, Ritskes-Hoitinga M, de Vries RBM, et al (2014) Systematic reviews of animal studies; missing link in translational research? PLoS ONE 9: e89981 10.1371/journal.pone.0089981 24670965 PMC3966727

[21] Bebarta V, Luyten D, Heard K (2003) Emergency medicine animal research: does use of randomization and blinding affect the results? Acad Emerg Med 10: 684–687. 12782533 10.1111/j.1553-2712.2003.tb00056.x

[22] van Wilgenburg E, Elgar MA (2013) Confirmation bias in studies of nestmate recognition: a cautionary note for research into the behaviour of animals. PLoS ONE 8: e53548 10.1371/journal.pone.0053548 23372659 PMC3553103

[23] Benignus VA (1993) Importance of experimenter-blind procedure in neurotoxicology. Neurotoxicol Teratol 15: 45–49. 8459788 10.1016/0892-0362(93)90044-o

[24] Tuyttens F, de Graaf S, Heerkens J, Jacobs L (2014) Observer bias in animal behaviour research: can we believe what we score, if we score what we believe? Anim Behav 90: 273–280.

[25] Ioannidis JPA (2005) Why most published research findings are false. PLoS Med 2: e124 16060722 10.1371/journal.pmed.0020124 PMC1182327

[26] Head ML, Holman L, Lanfear R, Kahn AT, Jennions MD (2015) The extent and consequences of p-hacking in science. PLoS Biol 13: e1002106 10.1371/journal.pbio.1002106 25768323 PMC4359000

[27] John LK, Loewenstein G, Prelec D (2012) Measuring the prevalence of questionable research practices with incentives for truth telling. Psychol Sci 23: 524–532. 10.1177/0956797611430953 22508865

[28] Simonsohn U, Nelson LD, Simmons JP (2014) p-curve and effect size: Correcting for publicaiton bias using only significant results. Perspectives on Psychological Science 9: 666–681. 26186117 10.1177/1745691614553988

[29] Cohen J (1988) Statistical power analysis for the behavioral sciences. 2nd ed Hillsdale, NJ: Erlbaum.

[30] Slatyer RA, Mautz BS, Backwell PRY, Jennions MD (2012) Estimating genetic benefits of polyandry from experimental studies: a meta-analysis. Biol Rev 87: 1–33. 10.1111/j.1469-185X.2011.00182.x 21545390

[31] Uller T, Nakagawa S, English S (2013) Weak evidence for anticipatory parental effects in plants and animals. J Evol Biol 26: 2161–2170. 10.1111/jeb.12212 23937440

[32] Prokop ZM, Michalczyk Ł, Drobniak SM, Herdegen M, Radwan J (2012) Meta-analysis suggests choosy females get sexy sons more than "good genes". Evolution 66: 2665–2673. 10.1111/j.1558-5646.2012.01654.x 22946794

[33] Trikalinos TA, Ioannidis J (2005) Assessing the evolution of effect sizes over time Publication Bias in Meta-Analysis: Prevention, Assessment and Adjustments. Chichester, UK: Wiley pp. 241–259.

[34] Jennions MD, Møller AP (2002) Relationships fade with time: a meta-analysis of temporal trends in publication in ecology and evolution. Proc Biol Sci 269: 43–48. 11788035 10.1098/rspb.2001.1832 PMC1690867

[35] Jennions MD, Lortie CJ, Rosenberg MS, Rothstein HR (2013) Publication and related biases Handbook of Meta-analysis in Ecology and Evolution. Princeton: Princeton University Press pp. 207–236.

[36] Low-Décarie E, Chivers C, Granados M (2014) Rising complexity and falling explanatory power in ecology. Frontiers in Ecology and the Environment 12: 412–418.

[37] Gelman A (2008) Scaling regression inputs by dividing by two standard deviations. Stat Med 27: 2865–2873. 17960576 10.1002/sim.3107

