# Supplementary figures and images for: Evidence of Experimental Bias in the Life Sciences: Why We Need Blind Data Recording
Source: PLoS Biol. 2015 Jul 8;13(7):e1002190. doi: 10.1371/journal.pbio.1002190 (PMC4496034; doi:10.1371/journal.pbio.1002190)

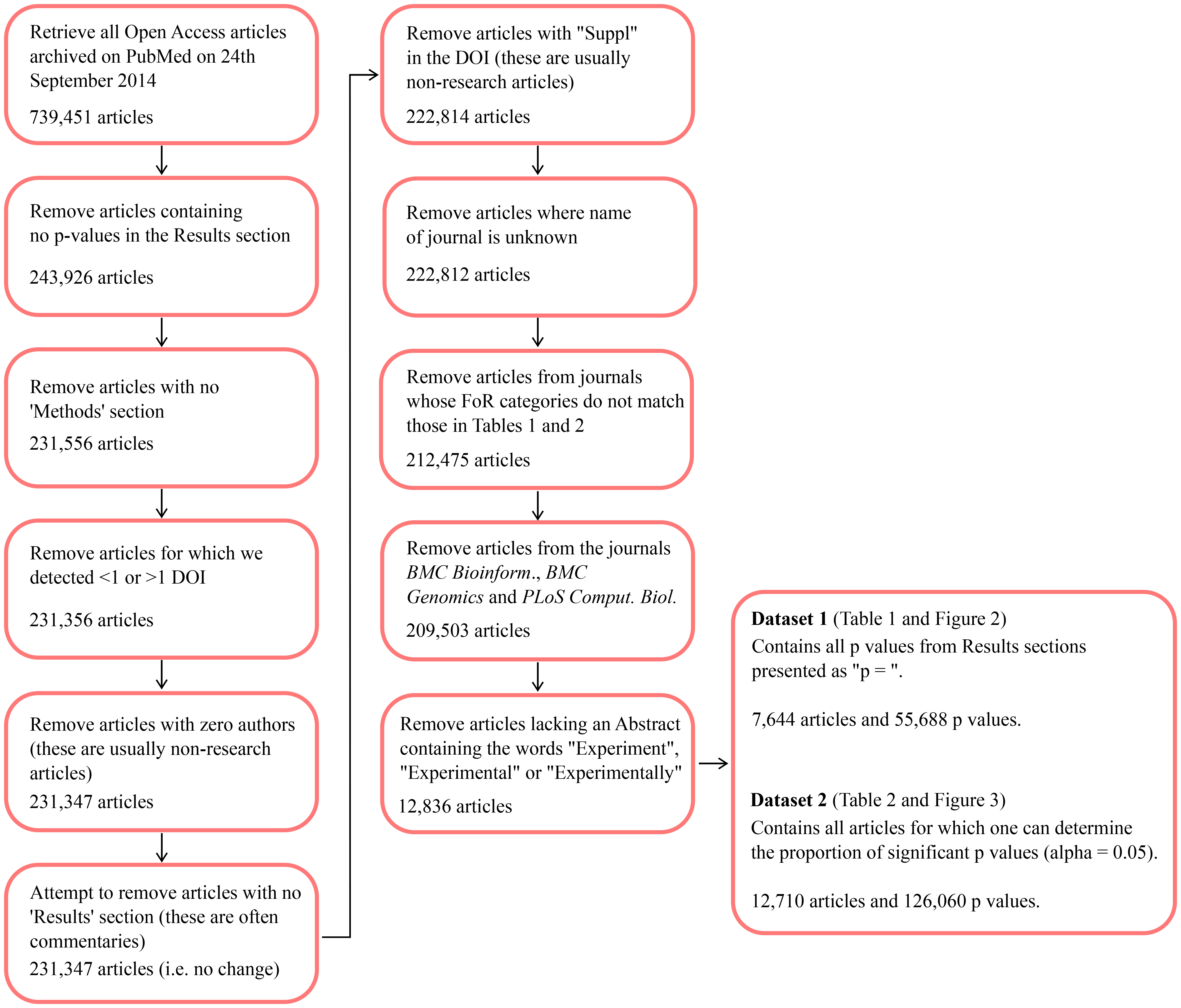

Supplement: S1 Fig — The flowchart shows how articles were culled from the initial list of 739,451 articles downloaded from PubMed to produce our two datasets. (TIF) [file pbio.1002190.s001.tif]

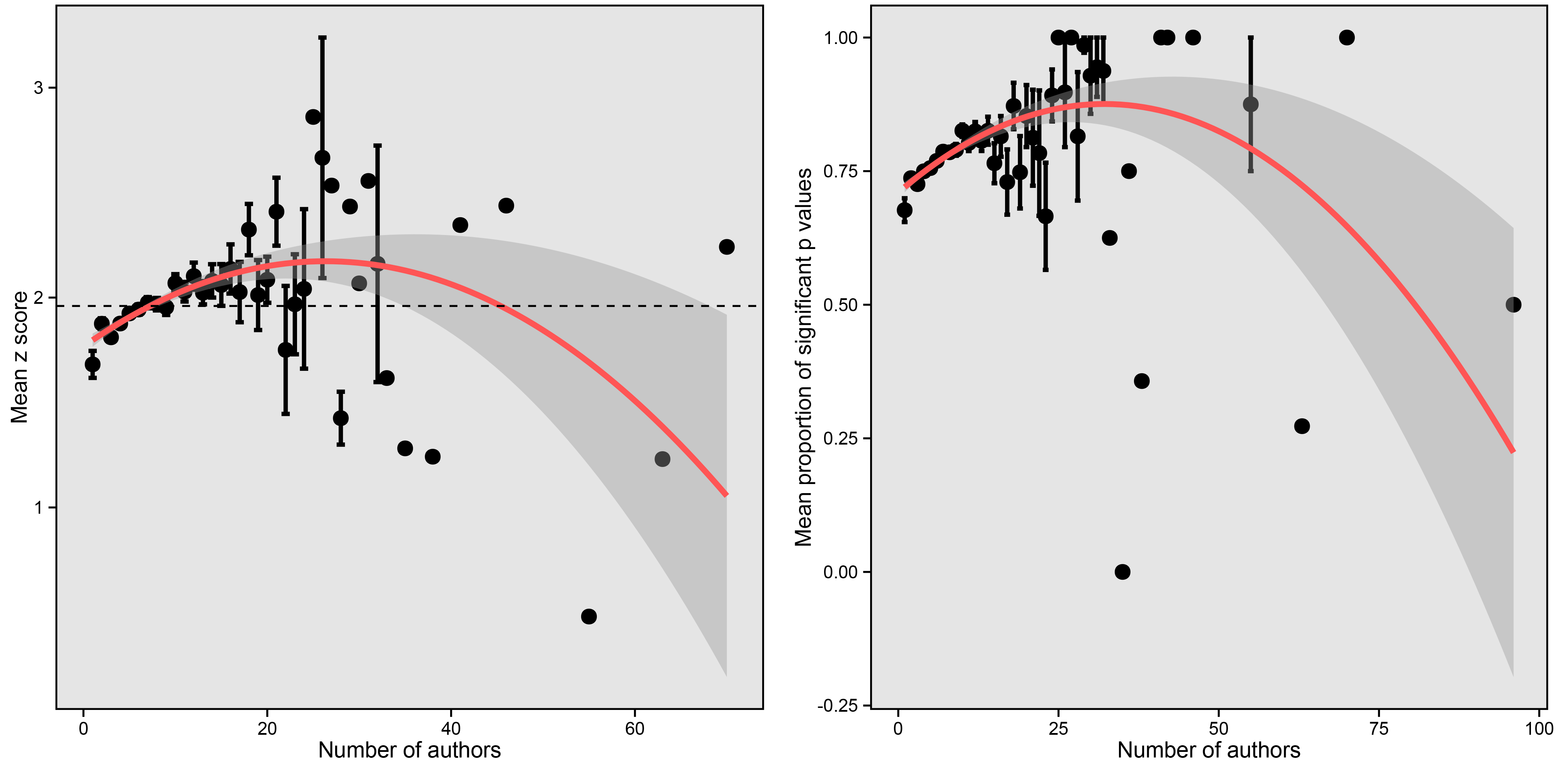

Supplement: S2 Fig — z scores and the proportion of significant p-values per paper increase with the number of authors on a paper, and this relationship levels off for higher author numbers. Both plots show means ± SE (points with no error bars have no replication); for the left panel, we first averaged all the z scores from each paper and then took the average of these for each year. The dashed line shows z = 1.96, which is equivalent to p = 0.05. Note that higher z scores denote lower p-values. (TIF) [file pbio.1002190.s002.tif]

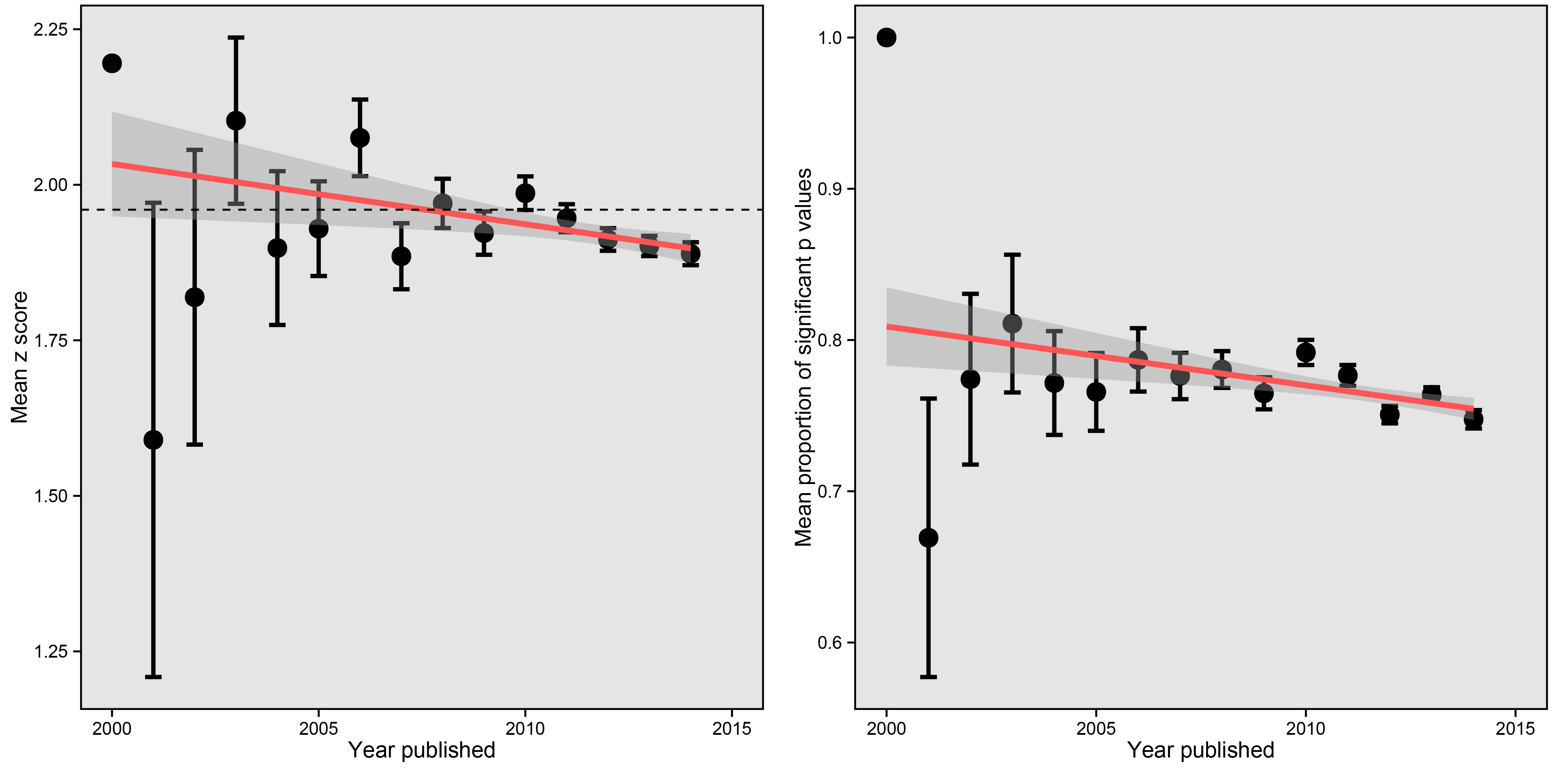

Supplement: S3 Fig — z scores and the proportion of significant p-values per paper have declined in recent years. Both plots show means ± SE (points with no error bars have no replication); for the left panel, we first averaged all the z scores from each paper and then took the average of these for each year. The dashed line shows z = 1.96. Note that higher z scores denote lower p-values. (TIF) [file pbio.1002190.s003.tif]

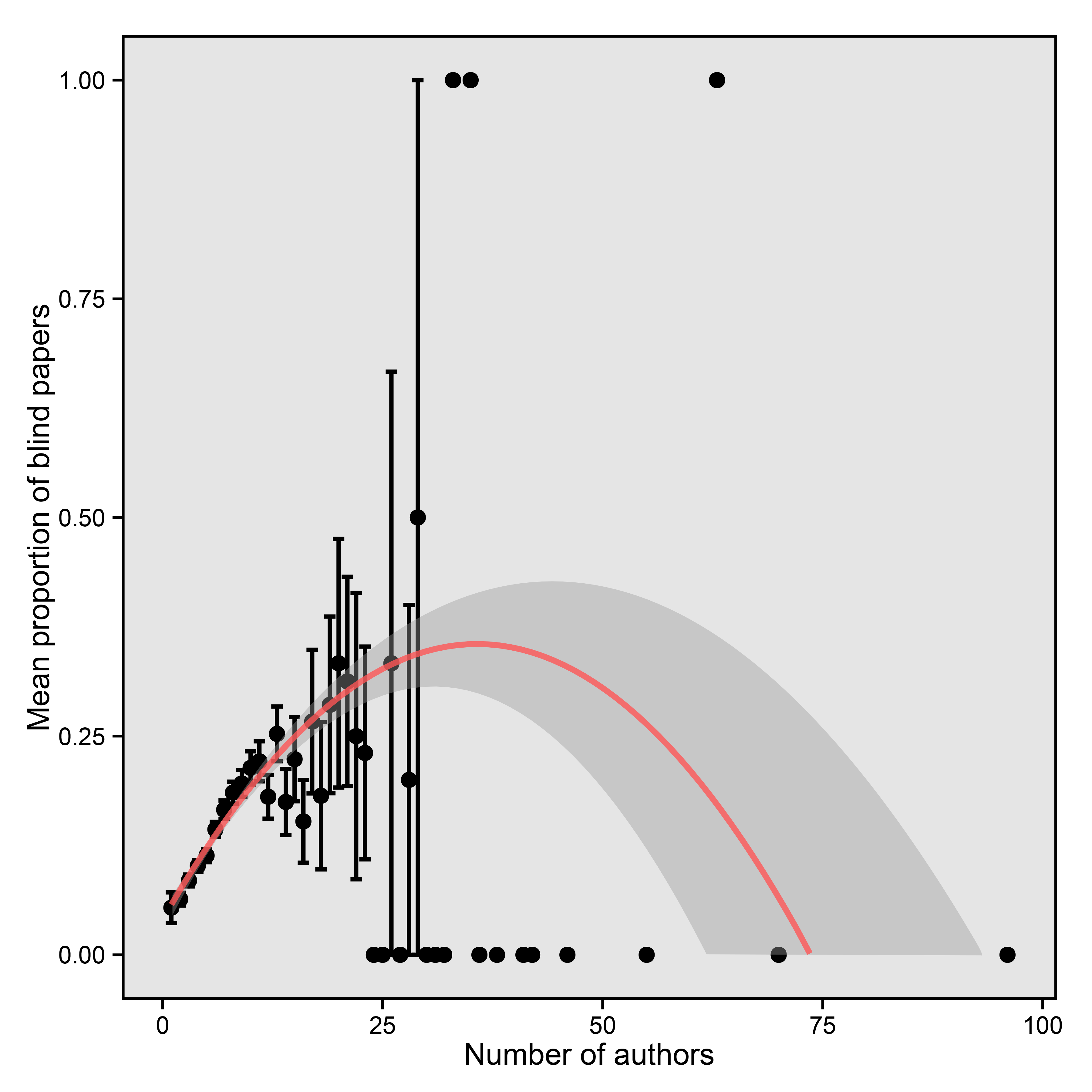

Supplement: S4 Fig — Effect of author number on the number of authors on a paper was positively correlated with the probability that a paper was blind, and this relationship began to level off for higher author numbers. Each point shows the mean ± SE for each number of authors (points with no error bars have no replication), and the line and its 95% CIs are from a quadratic regression on all the data. Note that quadratic regressions must invert at some point, but the paucity of data above c. 20 authors suggests that we have weak evidence that the relationship really does decline (not, for example, plateau) for high author numbers as suggested by the regression fit. (TIF) [file pbio.1002190.s004.tif]

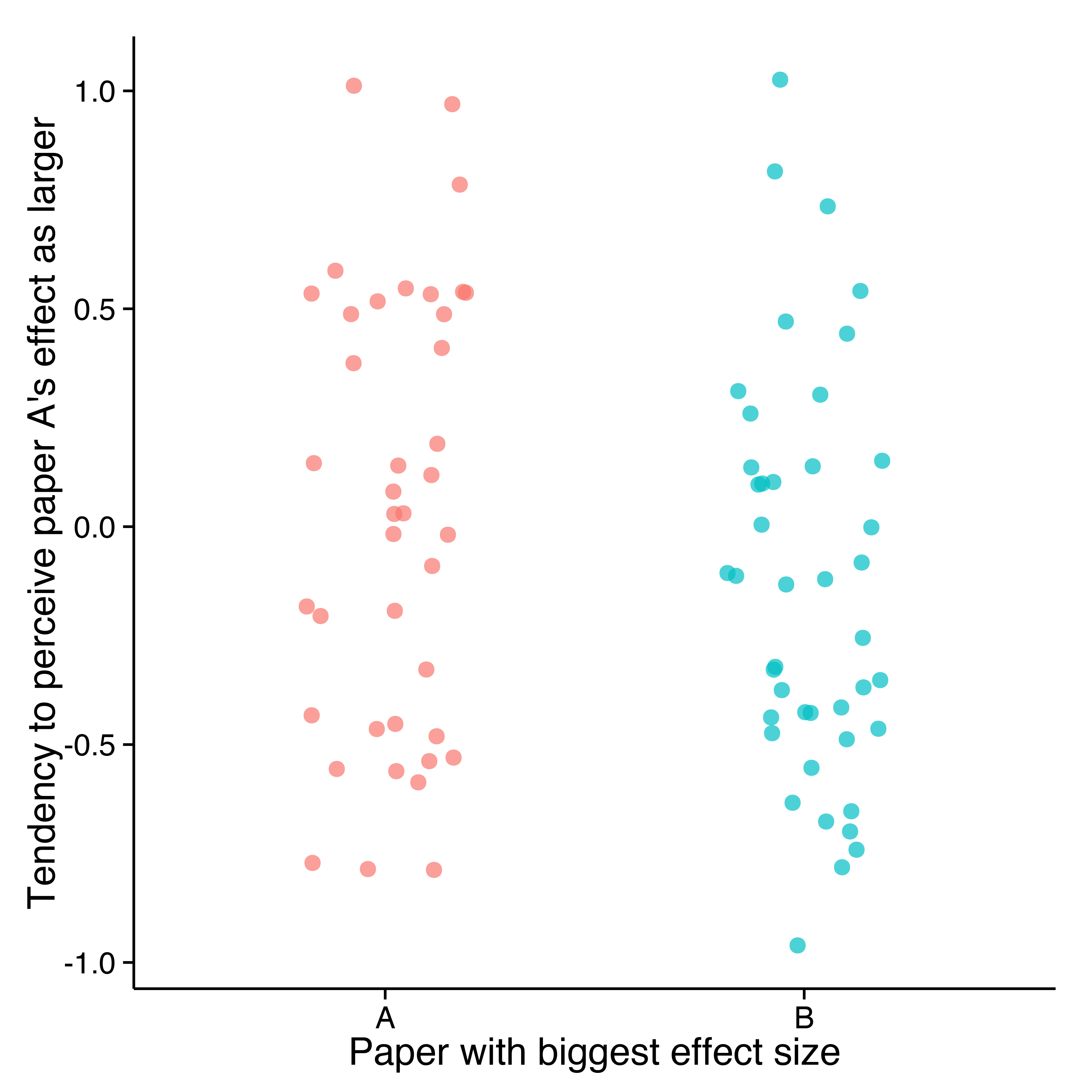

Supplement: S5 Fig — The x-axis shows which paper in the study pair had the larger absolute effect size (A and B are random names for the papers). The y-axis shows the tendency to think paper A had the larger effect, which was calculated as ((Number of participants thinking A was larger)–(Number thinking B was larger)) / 9. Therefore, a score of 1 means that 100% of participants thought A was larger, a score of -1 means that 100% thought B was larger, and zero means that the answers were evenly split. The distribution of answers was not affected by the true effect size difference of the papers (Mann-Whitney test: W = 1011, p = 0.17), and there are many study pairs in which the participants either disagreed about the answer or guessed incorrectly en masse. (TIF) [file pbio.1002190.s005.tif]
