# Supplementary material for: Evidence of Experimental Bias in the Life Sciences: Why We Need Blind Data Recording
Source: PLoS Biol. 2015 Jul 8;13(7):e1002190. doi: 10.1371/journal.pbio.1002190 (PMC4496034; doi:10.1371/journal.pbio.1002190)
Supplement: S1 Text — (DOCX) [file pbio.1002190.s006.docx]

# Online supplementary material

*From: Holman et al. 2015 PLoS Biology*

*Evidence of experimental bias in the life sciences: why we need blind data recording*

# Supplementary methods

**Literature search of effect sizes in blind and non-blind studies**

In short, we gathered a representative sample of blind experimental studies and paired these with a sample of non-blind experimental studies that asked comparable questions using a similar experimental design (where possible, in a related organism), then tested for a difference in effect size.

We focused on studies in our own field (evolutionary biology and related topics) so that we could appraise the studies accurately. Ninety-three blind studies were found by typing “observer was blind” and “performed blind” (with quotation marks) into Google Scholar in December 2013 and sorting by relevance. One of us (LH) went through the results and selected all papers whose titles suggested they might contain evolutionary biology experiments, and entered them into a spreadsheet. LH then checked all the papers to verify that they were in peer-reviewed journals, and that their Abstracts showed that they contained experimental biology (not non-empirical or correlational research). LH then read the selected papers’ methods to determine which result(s) were obtained via blind data recording, taking care not to look at the Results section or figures. When a result deriving from a blind experiment was found, LH wrote down the aim of the experiment that produced this result in the form of a general research question, e.g. “Does androgen treatment affect bird sexual traits?” or “Does CO_2_ affect anti-predator behavior in reef fish?”; these questions are archived on Data Dryad along with the raw data. Where there were multiple blind-collected results, one was selected with a random number generator. All LH’s questions mentioned the taxon of interest by its common name (with a haphazard level of specificity, e.g. “guppies” or “amphibians”), and did not state the direction or magnitude of the effect size found by the focal study.

To obtain matching non-blind studies, LH entered each research question into Google Scholar and sorted the results by relevance, then selected the first experimental study that asked a similar research question in a related taxon. LH did not have access to the blind papers' titles (or any other details) at this stage, and was thus unaware of their findings, still less their effect sizes. The non-blind papers were selected based on their similarity to the research question, i.e. without reference to their effect sizes (LH only read the title, and occasionally the abstract if the title was unclear). LH then checked whether the study was blind by reading the methods. If the study was blind, it was discarded and LH selected another study from the Google Scholar results until a non-blind paper was found. The number of blind studies thus discarded was recorded.

This search method has the advantage of easily locating many blind studies in spite of their rarity in the evolution literature, but it has the potential to create bias. Since the selection of studies was somewhat subjective, one may suspect that LH might have consciously or subconsciously selected studies that he inferred had larger and smaller effect sizes for the non-blind and blind paper sets, respectively. To evaluate the scope for such bias, we conducted a survey of nine professional evolutionary biologists, to ascertain whether papers’ titles and abstracts contain sufficient information to infer their effect sizes. The full methods and results are presented below, but in short, even the most successful survey participants were unable to gauge effect size from titles and abstracts. This result is expected, because the convention is for highly qualitative, prose-only abstracts in evolutionary biology, unlike other fields such as medicine (in which Abstracts commonly contain effect sizes). Therefore, the scope for bias at this stage of the study was minimal.

Next, LH handed the list of paired studies and their associated research questions to MLH, who at that time was unaware of the aim of this study or how the 93 paired studies had been chosen, preventing her expectations from biasing the outcome. The blind and non-blind studies’ positions in the spreadsheet were randomized, and where necessary the research questions were altered to obscure which study had been used to form the research question (e.g. if the blind study had been on mice but the paired non-blind study was on rats, the research question was amended to say “rodents” rather than “mice”). MLH then used the research questions to locate relevant data in the 93 pairs of papers, and calculated the standardized effect size (Hedges’ *g*, plus its variance) for each result using a function in the *compute.es* package for R appropriate to the type of data. The sign of the effect size was set as positive if the focal study found an effect in the predicted direction, and negative if it found an effect opposite to the predicted direction (the predicted direction was determined by reading the papers’ abstracts and introductions, as in [1], blind to the papers’ blindness). Thus, finding a higher average effect size in non-blind studies indicates that they are more likely to produce results in the predicted direction than are blind studies, consistent with observer bias.

We used meta-analysis, implemented in the R package *metafor* [2], to examine the effect sizes from the literature review. The *rma.mv* function was used to fit blindness as a moderator (fixed effect) and also as a random slope, and study pair was fitted as a random intercept. This approach assumes that study pairs differ randomly in their mean effect sizes, and that the effect of blindness on effect size varies randomly between study pairs around some mean value, which we are trying to estimate. The model was fitted using REML.

**Survey to examine the scope for bias during our literature review of evolutionary biology**

As mentioned in the main text, our search methods could have biased the results of the literature review of evolutionary biology, if is possible to infer differences in the effect sizes recorded by studies from their titles and abstracts. We thus conducted a survey (in February 2015) of 9 scientists with PhDs in evolutionary biology based at 4 research universities. The participants did not know the purpose of the survey. We presented them with the research questions (i.e. the ones written out by LH, available on Dryad), the papers’ titles, and the abstracts. Participants were asked to guess which paper had the higher effect size. Correct answers gave one point, passing was allowed (0 points), and incorrect answers resulted in deduction of one point. To ensure the participants were highly motivated to guess correctly, we offered a $100 prize for the participant who scored the most points.

The results of the quiz suggest that the titles and abstracts contain no information on effect size (Figure S5). The average score was 3.5 out of a possible 83 points (very close to zero, the score that indicates random guessing). After applying the Benjamini-Hochberg correction for multiple testing, not one of the 83 study pairs was guessed correctly more times than expected under the null hypothesis of random guessing by the nine participants (α = 0.05). Additionally, the top-scoring participant (score: 14 out of a possible 83), guessed correctly 48 times, incorrectly 34 times, and passed once. Thus, even the most accurate participant did not guess correctly more times than expected under the null hypothesis of random guessing (one-tailed binomial test: p = 0.09; note that strictly one should adjust this p value upwards to account for testing multiple participants). The results of the survey are not surprising, because abstracts in evolutionary biology rarely contain formal statistics, but instead discuss the results using qualitative prose (in our sample, just 1/166 papers had statistical results in the abstract).

Finally, one might suspect that if there were just a few study pairs for which the relative effect size was obvious, this would create enough bias to produce the effect of blindness we observed. We can identify candidates for such study pairs as those for which most participants guessed correctly. If our meta-analysis still suggests that blindness is important after these study pairs are cut out of the dataset, then the results are robust to any such bias introduced by our search method. If we cut out the 3 study pairs for which 9/9 people guessed correctly, or the 6 study pairs for which at least 8/9 people were correct, blindness still has a significant effect on effect size in our analysis (p = 0.022 and p = 0.032 respectively). It is only when we cut out the 15 study pairs for which at least 7/9 people guessed correctly that our results become non-significant, although there is still a trend in the predicted direction (p = 0.071). Thus, even if we throw away 14% of our putatively biased data, our results remain qualitatively unchanged (note that this is a conservative approach, because one expects several of the 83 study pairs to be guessed mostly correctly by chance alone).

**Text mining of p values from putatively blind and non-blind research**

We used text mining to search all open access papers available in the PubMed database. We first downloaded the Open Access subset of PubMed papers (available at <ftp://ftp.ncbi.nlm.nih.gov/pub/pmc/>) on 24^th^ September 2014, which compromised 870,962 papers from 4511 journals. For each of the available papers, we programmatically identified the Results section and extracted all numbers that appeared to be p values, i.e. numbers in the range 0-1 that were preceded by “p =”, “p <”, “p >”, “p ≤” or “p ≥” regardless of spacing or capitalization (for the precise regular expressions used, see [3]). We also recorded various details for each paper, including: (i) its DOI; (ii) the number of authors; (iii) year of publication; (iv) whether the abstract contained the words ‘experiment’, ‘experimental’, or ‘experimentally’ (regardless of capitalization); and (v) whether the Methods section contained the words or phrases ‘blind’, ‘blinded’ ‘blindly’, ‘not blind’, ‘not blinded’ or ‘not blindly’ (regardless of capitalization). Text mining was performed using a Python script available from ZENODO [3], which detected over 2.3 million p values in the 870,962 papers.

The Python script was efficient at retrieving p values. We showed that it found 98% of the p values that we were able to locate by manually searching 100 papers (729/744 p values), and that it rarely produced false positives: two numbers were erroneously designated as p values in these 100 papers (for details see [3]). An exception is p values from tables, figures and figure legends, which we were unable to collect because of their variable formatting. We were also unable to collect p values written in scientific notation, although these represented <1% of all p values in our manually-collected sample (7/744).

We then filtered and classified the papers in the initial dataset using an R script (see PRISMA chart in Figure S1). To maximize the representation of experimental research papers in our dataset, and minimize the presence of papers such as editorials, reviews and theoretical models (which do not require blind protocols), we only included papers in the dataset if they 1) had an Abstract, Methods and Results section, 2) had at least one of the words “experiment”, “experimental” or “experimentally” in the abstract, 3) had at least one author, or 4) did not have the character string “Suppl” in the DOI (inspection revealed that these were generally not research papers). Additionally, we discounted papers that contained no detectable p values from all analyses. In the same R script, we classified each paper as blind or not blind. Papers were classified as blind if their Methods section contained at least one of the words “blind”, “blinded” or “blindly”, and did not contain any of the phrases “not blind”, “not blinded” or “not blindly”.

Each paper was also classified into a subject area based on the journal in which it was published. We classified journals into subject areas based on their “Field of Research” (FoR) code in the Australian and New Zealand Standard Research Classification system, as previously described [4]. Initial investigations revealed that in our dataset, some FoR categories (namely Environmental Sciences, Mathematical Sciences, Earth Sciences, Information and Computing Sciences, and Education) contained fewer than 10 papers that were classified as blind. We elected to remove these categories from the dataset, as well as the journals *BMC Bioinformatics,* *BMC Genomics* and *PLoS Computational Biology* (which were well-represented in our initial dataset but contained almost no blind papers), since a near-total absence of blindness in a field or journal implies that blindness is rarely necessary.

Text mining on this scale is always imperfect. We therefore manually checked 200 randomly selected papers that made it into our dataset, 100 of which had been classified as blind, and 100 as not blind. These papers were then thoroughly read to verify whether they were truly blind or not. For both sets of papers, the misclassification rate was 5% (5/100), e.g. because a paper that we had classified as blind was actually explaining that it was not blind, or because a paper that we had classified as non-blind had declared that it was blind using roundabout language. We were wary of “single blind” studies, because these would contain the word “blind” despite (usually) being conducted by researchers who were not blind. However, we found no single blind studies in the 100 blind-classified papers, suggesting such papers are rare in our dataset. We amended the blindness classifications for the 10 errors in our final dataset (i.e. the one archived on Dryad). While the remaining misclassification errors will add noise to our estimates of the effect of blindness on research outcomes, they should not systematically bias our findings in any particular direction.

**Statistical analysis of text-mined p values**

For the first analysis, we examined all of the exact p values (i.e. those preceded by an equals sign) that were returned by text mining. These p values were first transformed to 2-tailed z scores using the *qnorm* function in R as follows: *z* = *qnorm*(1 - (*p*/2)), to better approximate a normal distribution. To facilitate transformation, p values that were written as p = 0 were first changed to p = 0.0001.

The z scores were analysed with linear mixed models using paper ID as a random factor, to account for the possible similarity of p values derived from the same paper. In the full model, we fitted blindness and FoR category (plus their interaction), year of publication, the number of authors, and the quadratic term for number of authors (since the relationship with z score appeared to be non-linear; see Figure S1). To facilitate model comparison, models were calculated using maximum likelihood rather than REML [5]. We then ranked all possible models from the full model to the null model (total = 30 models) by their AICc scores using the *dredge* function in the *MuMIn* package for R [6]. Since several models had similarly good AICc scores, we estimated each parameter’s effects using model averaging (via the *model.avg* function in *MuMIn*) on all models with an Akaike weight >0.05 [e.g. 7,8].

We also constructed a dataset that listed the number of p values in each paper that were significant and non-significant (α = 0.05). Certain p values are ambiguous for this classification (e.g. p = 0.05, p > 0.03 and p ≤ 0.05), and were excluded. In contrast to the previous analysis (which used exact p values only, and not p values presented as inequalities), this analysis allowed us to use many more of the p values that were detected during text mining. Since the response variable was binomial and we detected over-dispersion, we used generalized linear models with quasibinomial errors (response variable: a two-column vector of the number of significant and non-significant p values in each paper). We fitted the same set of models as for the z score dataset, and ranked them as before (except that we ranked models by their QAIC scores, since the models used quasi-likelihood). Model averaging was not needed because the top model had a high Akaike weight [7].

# Supplementary references

1. Bello S, Krogsbøll LT, Gruber J, Zhao ZJ, Fischer D, et al. (2014) Lack of blinding of outcome assessors in animal model experiments implies risk of observer bias. J Clin Epidemiol 67: 973–983.

2. Viechtbauer W (2010) Conducting meta-analyses in R with the metafor package. J Stat Software 36: 1–48.

3. Lanfear R (2014) pvalues version 0.1. 10.5281/zenodo.13147: 10.5281/zenodo.13147.

4. John LK, Loewenstein G, Prelec D (2012) Measuring the prevalence of questionable research practices with incentives for truth telling. Psychol Sci 23: 524–532.

5. Zuur A, Ieno EN, Walker N, Saveliev AA, Smith GM (2011) Mixed Effects Models and Extensions in Ecology with R. New York: Springer.

6. Bartoń K (2012) MuMIn: multi-model inference. R package version 1-10-5.

7. Anderson DR (2007) Model Based Inference in the Life Sciences. New York: Springer.

8. Grueber CE, Nakagawa S, Laws RJ, Jamieson IG (2011) Multimodel inference in ecology and evolution: challenges and solutions. J Evol Biol 24: 699–711.
